# Lipid profile in Noonan syndrome and related disorders: trend by age, sex and genotype

**DOI:** 10.3389/fendo.2023.1209339

**Published:** 2023-07-31

**Authors:** Federica Tamburrino, Laura Mazzanti, Emanuela Scarano, Dino Gibertoni, Maria Sirolli, Maximiliano Zioutas, Concetta Schiavariello, Annamaria Perri, Alessio Mantovani, Cesare Rossi, Marco Tartaglia, Andrea Pession

**Affiliations:** ^1^ Rare Diseases Unit, Department of Pediatrics, IRCCS Azienda Ospedaliero-Universitaria Di Bologna, Bologna, Italy; ^2^ Alma Mater University of Bologna, Bologna, Italy; ^3^ Research and Innovation Unit, IRCCS Azienda Ospedaliero-Universitaria Di Bologna, Bologna, Italy; ^4^ Pathology Unit, Department of Experimental, Diagnostic and Specialty Medicine, IRCCS Azienda Ospedaliero-Universitaria Di Bologna, Bologna, Italy; ^5^ Department of Pediatrics, IRCCS Azienda Ospedaliero-Universitaria Di Bologna, Bologna, Italy; ^6^ Medical Genetics Unit, IRCCS Azienda Ospedaliero-Universitaria Di Bologna, Bologna, Italy; ^7^ Molecular Genetics and Functional Genomics, Ospedale Pediatrico Bambino Gesù, IRCCS, Rome, Italy

**Keywords:** Noonan Syndrome, Mazzanti syndrome, RASopathies, cholesterol, tryglicerides, BMI, *PTPN11*, *SHOC2*

## Abstract

**Background:**

RASopathies are developmental disorders caused by dysregulation of the RAS-MAPK signalling pathway, which contributes to the modulation of multiple extracellular signals, including hormones and growth factors regulating energetic metabolism, including lipid synthesis, storage, and degradation.

**Subjects and methods:**

We evaluated the body composition and lipid profiles of a single-centre cohort of 93 patients with a molecularly confirmed diagnosis of RASopathy by assessing height, BMI, and total cholesterol, HDL, triglycerides, apolipoprotein, fasting glucose, and insulin levels, in the context of a cross sectional and longitudinal study. We specifically investigated and compared anthropometric and haematochemistry data between the Noonan syndrome (NS) and Mazzanti syndrome (NS/LAH) groups.

**Results:**

At the first evaluation (9.5 ± 6.2 years), reduced growth (-1.80 ± 1.07 DS) was associated with a slightly reduced BMI (-0.34 DS ± 1.15 DS). Lipid profiling documented low total cholesterol levels (< 5^th^ percentile) in 42.2% of the NS group; in particular, in 48.9% of *PTPN11* patients and in 28.6% of NS/LAH patients compared to the general population, with a significant difference between males and females. A high proportion of patients had HDL levels lower than the 26^th^ percentile, when compared to the age- and sex-matched general population. Triglycerides showed an increasing trend with age only in NS females. Genotype-phenotype correlations were also evident, with particularly reduced total cholesterol in about 50% of patients with *PTPN11* mutations with LDL-C and HDL-C tending to decrease during puberty. Similarly, apolipoprotein A1 and apolipoprotein B deficits were documented, with differences in prevalence associated with the genotype for apolipoprotein A1. Fasting glucose levels and HOMA-IR were within the normal range.

**Conclusion:**

The present findings document an unfavourable lipid profile in subjects with NS, in particular *PTPN11* mutated patients, and NS/LAH. Further studies are required to delineate the dysregulation of lipid metabolism in RASopathies more systematically and confirm the occurrence of previously unappreciated genotype-phenotype correlations involving the metabolic profile of these disorders.

## Introduction

Dysregulation of the RAS-MAPK pathway contributes to the control of metabolism and energy storage (1). Energy metabolism is consistently found to be dysregulated in RASopathies (2–6), which constitute a family of disorders caused by mutations in genes encoding key transducers participating in the RAS-MAPK signalling cascade (7, 8). Metabolic and nutritional aspects in RASopathies are, however, still poorly explored (9). Feeding difficulties are well recognized to represent a relevant concern in these disorders, especially during infancy and in association with specific genotypes (10, 11). A reduction of adiposity has been documented in patients with Noonan syndrome (NS; MIM: PS163950) (12) and LEOPARD syndrome (also known as Noonan syndrome with multiple lentigines) (NSML; MIM: PS151100) (13) and a particularly low BMI score has been reported in Mazzanti syndrome (also known as Noonan-like disorder with loose anagen hair) (NS/LAH; MIM: 607721) (14). A recent study on body composition in RASopathies suggests the occurrence of simultaneous impairment in adipose and muscle mass tissues (9).

Few data are available on the metabolic and lipid profiles characterizing these disorders. Based on these considerations, the aim of this work was to evaluate the fasting lipid profile, in particular cholesterol and triglycerides levels, in a large and unselected single-centre cohort of patients affected by a RASopathy with a molecularly confirmed diagnosis, who had been in follow-up from childhood until early adulthood.

## Patients and methods

A retrospective longitudinal study was performed involving 93 patients with clinical features fulfilling the criteria for RASopathies and whose diagnosis had been confirmed by molecular analysis.

The patients were recruited at the Paediatric Rare Disease Outpatient Unit of *IRCCS Azienda Ospedaliero-Universitaria di Bologna*, Italy, from 2001 to 2020, and were followed until April 2022.

Molecular analyses were performed by parallel sequencing using a continuously updated panel of genes implicated in these disorders, as previously reported (14). For a subset of patients, the molecular diagnosis had been achieved by means of targeted Sanger sequencing.

A fasting blood sample was taken every six months or yearly from each patient. The following parameters were analyzed: total cholesterol (TC), HDL cholesterol (HDL-C), triglycerides (TG). LDL cholesterol (LDL-C) blood levels were calculated using the Friedewald formula (LDL cholesterol = total cholesterol – HDL cholesterol – triglycerides/5) (15).

Among the different RASopathies belonging to this cohort, here we present, discuss and compare the anthropometric and haematochemistry data collected in the NS and the NS/LAH groups.

The study was conducted according to the Declaration of Helsinki and Good Clinical Practice guidelines and was approved by the Emilia-Romagna AVEC ethics committee [internal code 119/2022/Oss/AOUBo].

### Statistical analysis

Height and body mass index (BMI, Kg/m^2^) were expressed as standard deviation scores (SDS) and referred for age- and sex-specific groups. Anthropometric measurements were compared to the standard growth curves for the general Italian population (16).

Blood concentrations of TC and lipoproteins in patients between 12 and 20 years of age were compared to the corresponding age and sex standards of the “National Health and Nutritional Examination Surveys” (17). Specifically, we used the general population 5^th^ percentile as a reference point for low levels of TC and LDL, the 26^th^ percentile for low levels of HDL-C, and the 54^th^ percentile for above normal values of LDL.

Apolipoprotein levels were compared to the sex-matched reference ranges determined by using the same in-house analytical procedures (Apo A1, 105-205 mg/100ml Apo B 55-130 mg/100ml [females]; Apo A1, 105-175 mg/100 ml, ApoB, 60-140 mg/100ml [males]).

The distributions of lipid profile biomarker levels (TC, HDL-C, LDL-C and TG) were compared across genotypes using the Mann-Whitney test. The trajectories of those biomarkers for age and sex were obtained as margins (linear predictions) from regression models in which the biomarker was the dependent variable, and the interaction of sex and age (in rounded years) was the predictor. Data collected at all ages were used in these regressions, and standard error correction was included to account for repeated data from the same patients.

The proportions of biomarkers with values below or above the standard cutoffs were compared across sexes and genotypes using chi-square or Fisher’s exact test.

All analyses were performed using Stata v.17.0; p-values lower than 0.05 were considered statistically significant.

## Results

The study cohort included 93 patients, 53 (57.0%) males and 40 (43.0%) females; at the first evaluation mean age was 9.5 ± 6.2 years (min 1.5 – max 27.2). Clinically, the majority of patients (N=70, 75.3%) had a diagnosis of NS; 10 (10.7%) subjects were diagnosed with NS/LAH, while 5, 4, and single patients had a diagnosis of cardio-facio-cutaneous syndrome (CFCS; MIM: PS115150) (5.4%), NSML (4.3%), Costello syndrome (MIM: 218040, Legius Syndrome (MIM: 611431), *MAPK1*-related syndrome (MIM: 619087) and *CBL*-related syndrome (MIM: 613563) (1.1%), respectively (**Table 1**). Among the NS patients, 49 (70.0%) had *PTPN11* mutations, while 21 patients were non-*PTPN11* and each of the other genotypes accounted for less than 10%. In the NS/LAH group of patients, 9 out of 10 had a *SHOC2* mutation, the remaining having a pathogenic missense change in *PPP1CB*.

**Table 1:** List and frequency of genotypes in the studied RASopathy cohort.

Genotype	Entire Cohort	
	n	%
Noonan syndrome	70	75.3
*PTPN11*	49	52.7
*SOS1*	6	6.4
*LZTR1*	5	5.4
*RAF1*	4	4.3
*RIT1*	3	3.2
*KRAS*	2	2.1
*SOS2*	1	1.1
Noonan syndrome with multiple lentigines		
*PTPN11*	4	4.3
Cardiofaciocutaneous syndrome	5	5.4
*BRAF*	2	2.1
*KRAS*	2	2.1
*MEK1*	1	1.1
Costello syndrome		
* HRAS*	1	1.1
Mazzanti syndrome	10	10.7
*SHOC2*	9	9.7
*PPP1CB*	1	1.1
Legius syndrome		
*SPRED1*	1	1.1
*MAPK1*-related syndrome *MAPK1*	1	1.1
*CBL*-related syndrome *CBL*	1	1.1
Total	93	100.0

At the first evaluation, our patients were shorter than the general population overall (-1.80 ± 1.07 DS) and had a slightly lower BMI (-0.34 DS ± 1.15 DS). Stature was lower in NS/LAH (-2.76 SDS) compared to the general NS group (-1.69 SDS; p=0.004) and *PTPN11*-related NS subgroup (-1.81 SDS; p=0.015). BMI was not significantly lower in NS/LAH (-0.73 SDS) compared to the NS group (-0.17 SDS; p=0.183) and *PTPN11*-related NS subgroup (-0.38 SDS; p=0.450). Correlations between BMI and TC, cholesterol fractions, TG were almost null (from 0.021 to 0.106). BMI showed only a mild positive correlation with TG in males (r= 0.322).

### Lipid profile

Considering all the blood samples collected in the NS and NS/LAH patient groups, TC levels were significantly higher in females than males (**Table 2**): 156.0 ± 31.2 vs. 137.6 ± 29.5 mg/dL (p<0.001) in NS patients and, in particular, 152.5 ± 32.6 vs. 136.1 ± 27.1 mg/dL (p=0.001) in the NS subgroup with mutated *PTPN11*, and 160.8 ± 34.3 vs. 133.5 ± 18.7 mg/dL (p=0.013) in NS/LAH patients. Also, LDL-C and TG were higher in females, while HDL-C values were similar for both sexes (**Table 2**).

**Table 2:** Comparison of lipid profile of males and females in patients with Noonan syndrome and Mazzanti syndrome (n=80).

	Males (47 patients; 161 observations)	Females (33 patients; 97 observations)	test; p-value
Age (years)	12.5 ± 7.0	12.2 ± 8.2	-0.93; 0.351
Total cholesterol (TC) (mg/dl)	137.6 ± 28.5	156.0 ± 31.5	4.92; <0.001
HDL cholesterol ((mg/dl)	45.6 ± 12.2	45.4 ± 13.0	-0.05; 0.958
LDL cholesterol (mg/dl)	80.4 ± 25.2	99.0 ± 29.9	5.08; <0.001
Triglycerides (mg/dl)	60.1 ± 24.6	78.7 ± 36.0	4.68; <0.001
HDL/TC	0.34 ± 0.08	0.29 ± 0.07	-3.79; <0.001
HDL/LDL	0.62 ± 0.24	0.49 ± 0.16	-3.94; <0.001

**Figure 1:**
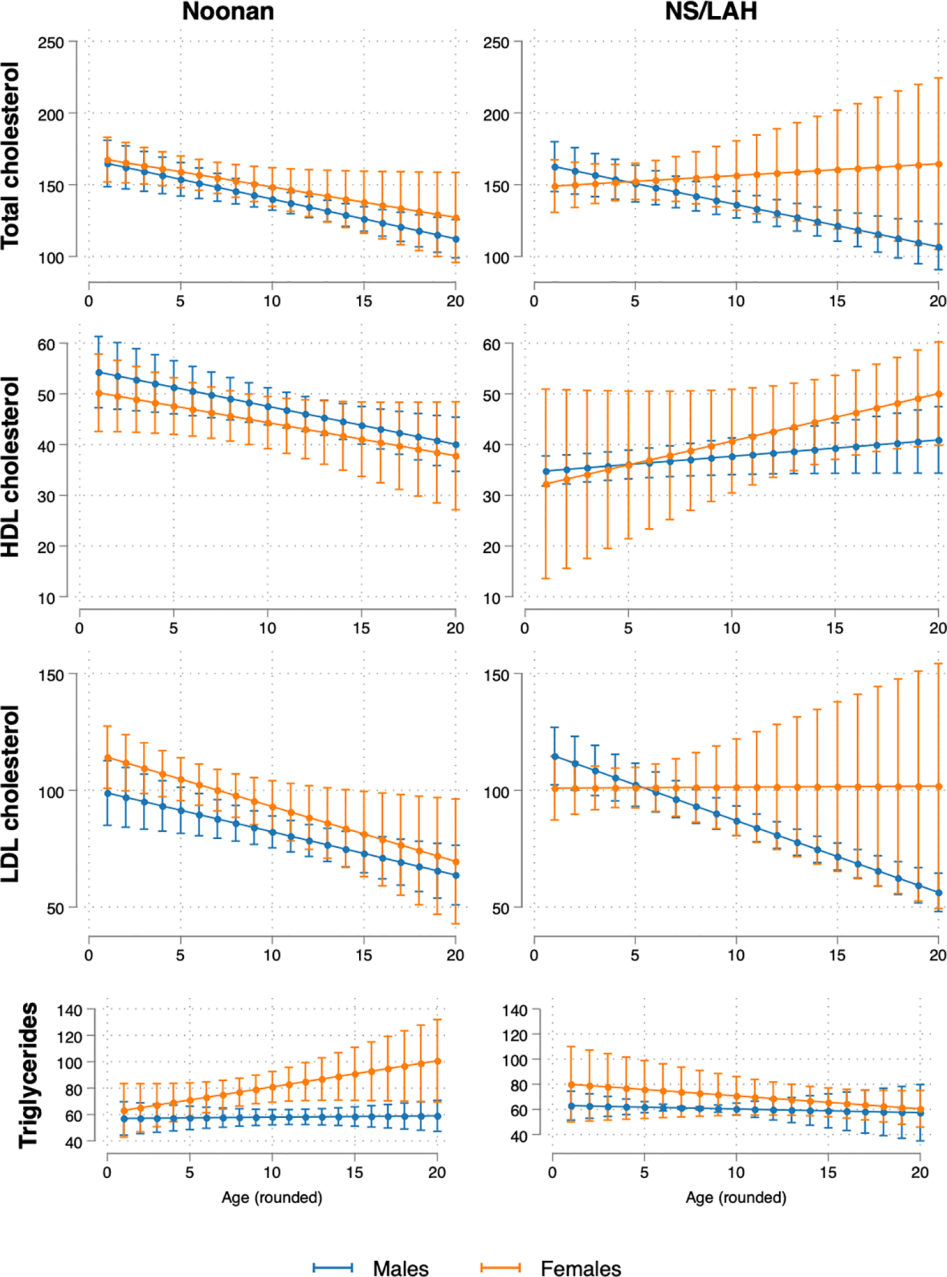
Model-estimated mean values and 95% CI of lipid profile biomarkers (TC, HDL-C, LDL-C and triglycerides) by age (1-20 years) and sex, for NS and NS/LAH patients.

The estimated trajectories of TC, HDL-C and LDL-C decreased with age in NS patients, with similar trends seen in both males and females. In NS/LAH, values decreased with age in males and were stable or slightly increased in females, although few subjects were evaluated. Triglycerides showed an increasing trend with age in NS females and a stable trend in NS males and in NS/LAH (**Figure 1**).

When comparing our patients affected by NS and NS/LAH with the general population, we tested all the blood samples collected in the 12 to 20-year age range (**Table 3**).

**Table 3:** Comparison of the lipid profile between patients with Noonan syndrome, Mazzanti syndrome and PTPN11 genotype and the general population.

	TC < 5^th^ percentile (%)	HDL < 26^th^ percentile (%)	LDL < 5^th^ percentile (%)	LDL borderline-high (%)
NS and NS/LAH females (n=33)	38.5	53.8	34.6	34.6
NS and NS/LAH males (n=47)	40.4	48.1	11.5	16.1
test females vs. males	0.03; 0.870	0.23; 0.631	0.12; 0.725	5.94; 0.015
NS (30 pts, 64 obs.)	42.2	54.7	34.4	17.2
NS/LAH (7 pts, 14 obs.)	28.6	28.6	7.1	28.6
*PTPN11*-NS (21 pts, 45 obs)	48.9	62.2	37.8	17.8
non-PTPN11 NS (9 pts, 19 obs.)	26.3	36.8	26.3	15.8
test NS vs. NS/LAH	0.386^	0.138^	0.054^	0.453^
test NS/LAH vs. *PTPN11-NS*	0.227^	0.035^	0.044^	0.453^
test *PTPN11-NS* vs. non-PTPN11 NS	2.79; 0.095	3.47; 0.062	0.78; 0.378	1.000^

chi-square test, except:

^= Fisher’s exact test.

Observations from patients in the 12-20 age range are included.

**Figure 2:**
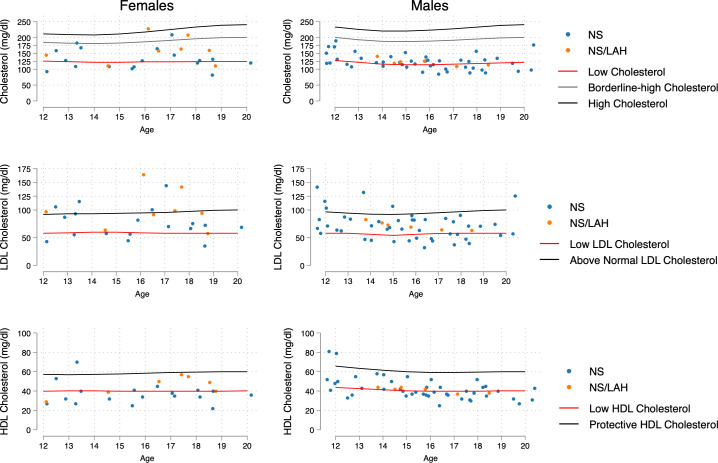
Distribution of age- and sex-related values of TC, LDL-C and HDL-C, compared with increased-risk curves for the general population (age 12-20 years) taken from Joliffe C.J. and Janssen I (17). The 5^th^ percentile was the reference point for low levels of TC and LDL, the 26^th^ percentile for low levels of HDL-C, and the 54^th^ percentile for above normal values of LDL.

The lipid profile was characterized by TC values lower than the 5^th^ percentile for the general population in 42.2% of the NS group (41.3% in males vs 44.4% in females, p=0.819); in particular, in 48.9% of the *PTPN11*-related NS subgroup (46.7% in males vs. 53.3% in females, p=0.673), in 26.3% of the non-*PTPN11* NS subgroup (31.2% in males vs. 0% in females, p=0.530), and in 28.6% of NS/LAH patients (33.3% in males vs. 25.0% in females, p=0.594) (**Figure 2**).

Females showed a significantly higher percentage of above normal LDL-C levels than males when considering the NS and NS/LAH groups (34.6% vs 11.5%, p=0.015). The proportions of low LDL-C values under the 5^th^ percentile differed significantly in NS/LAH compared to *PTPN11* mutated patients (p=0.044), with borderline significance in NS compared to NS/LAH patients (p=0.054) (**Table 3**).

When comparing the lipid profiles of the different genotypes, NS patients showed higher levels of HDL-C than NS/LAH patients (p=0.046) and a higher ratio of HDL/LDL (p=0.035) (**Table 4**).

**Table 4:** Comparison of the lipid profile between patients with Noonan syndrome, and the *PTPN11* genotype subgroup to Mazzanti syndrome subjects.

	Mean ± standard deviation				Test; p-value		
	NS (n=70, 227 observations)	NS/LAH (n=10, 31 observations)	*PTPN11*NS (n=49, 169 observations)	non*PTPN11* NS (n=21, 58 observations)	NS vs. NS/LAH	NS/LAH vs. *PTPN11*NS	*PTPN11*NS vs. non*PTPN11*NS
Age (years)	12.3 ± 7.5	13.5 ± 6.8	11.9 ± 7.3	13.5 ± 8.2	-1.16; 0.245	-1.52; 0.129	-1.07; 0.283
TC (mg/dl)	144.3 ± 31.2	144.4 ± 28.6	142.5 ± 30.4	149.7 ± 33.1	0.08; 0.937	-0.45; 0.654	-1.30; 0.193
HDL-C (mg/dl)	46.1 ± 12.8	40.6 ± 8.9	46.3 ± 12.7	45.5 ± 13.0	1.99; 0.046	1.83; 0.067	0.71; 0.476
LDL-C (mg/dl)	86.7 ± 28.8	90.9 ± 25.3	85.0 ± 27.9	91.9 ± 31.0	-0.86; 0.391	-1.37; 0.171	-1.42; 0.155
TG (mg/dl)	67.6 ± 31.9	63.7 ± 19.4	66.9 ± 30.2	69.4 ± 37.0	-0.12; 0.907	-0.17; 0.865	-0.29; 0.770
HDL/TC	0.33 ± 0.08	0.29 ± 0.06	0.33 ± 0.08	0.31 ± 0.09	1.81; 0.071	2.17; 0.030	1.81; 0.070
HDL/LDL	0.59 ± 0.23	0.48 ± 0.13	0.60 ± 0.22	0.56 ± 0.24	2.11; 0.035	2.49; 0.013	1.73; 0.084

Mann-Whitney test, except:

^= Fisher’s exact test.

* = chi-square test.

### Lipid profile and puberty

Lipid profiles were obtained from assessments of 90 prepuberal females (mean age 11.6 ± 8.5), 116 prepuberal males (mean age 9.6 ± 6.2), 29 pubertal females (mean age 17.6 ± 4.7) and 61 pubertal males (mean age 17.5 ± 4.9). Significant differences between prepubertal and pubertal assessments, with lower values in puberty, were found in males for TC (127.2 vs. 141.7 mg/dl in puberty vs. pre-puberty, p<0.001) and LDL-C (73.7 vs. 83.8 mg/dl, p=0.010). A similar pattern was found for LDL-C in females (88.6 vs. 102.4 mg/dl, p=0.053). However, no significant differences between prepubertal and pubertal assessments was found in the proportion of subjects with TC, LDL-C, HDL-C and TG values below the lowest threshold. The same pattern of significantly lower TC and LDL-C values in pubertal compared to prepuberal assessments was found also when analysing NS patients and NS/LAH separately. In NS patients, a significant difference was also found for HDL-C, again with lower values in puberty (42.4 vs. 47.6 mg/dl, p=0.005).

### Apolipoproteins

Apolipoprotein A1 (ApoA1) and B (ApoB) concentrations were only tested in 12/93 patients, among which 9/12 had NS and 3/12 NS/LAH. Thirty-three percent of patients showed ApoA1 deficiency and 33% ApoB deficiency. ApoA1 deficiency was observed in 2/9 (22.2%) NS patients and in 2/3 (66.7%) NS/LAH patients. ApoB deficiency was documented in 3/9 (33.3%) NS patients and in 1/3 (33.3%) NS/LAH patients.

### Glucose metabolism

Blood glucose levels were nearly significantly higher in NS patients than in NS/LAH subjects (mean values: 80.7 vs. 76.0, p = 0.060). HOMA IR levels did not differ significantly (1.47 vs. 1.37, p = 0.758). In both groups, the mean values were within the normal range for the general population.

## Discussion

In this study, we characterize the lipid profile in a large and unselected cohort of patients with RASopathies.

In the general population, a physiological fluctuation in lipoprotein levels during adolescence and young-adult age has been reported. In particular, Jolliffe and Janssen (17) elaborated curves corresponding to borderline high and high levels for TC and LDL and for low and protective HDL-C. Our data provide evidence that patients with NS and NS/LAH show significant differences in their lipid profile compared to the general population in the 12 to 20-year age range, according to age and sex. Specifically, TC values were lower than the 5^th^ percentile for the general population standard range in 42.2% of the NS group (in particular, in 48.9% of patients carrying a mutated *PTPN11* allele) and in a lower percentage of the NS/LAH group (28.6%).

In relation to the values trend for age and gender, Joliffe and Janssen (17) reported that in both sexes TC concentration declines during the early pubertal period and subsequently increases to reach adult levels, while LDL-C and HDL-C present a different trajectory in males and females. In males, LDL-C decreases in early adolescence and then increases from 15.5 years of age, instead, in females, there is a regular increase until adulthood. HDL-concentration in males declines moderately until 16 years of age and then stabilizes, whereas females do not show any modification during the pubertal period.

Even in our cohort, lipid profiles differed by sex, as females had significantly higher TC, LDL-C and TG levels than males in all the groups of patients evaluated, although they remain within the normal range for the general population.

As shown in **Figure 1**, in puberty, differently from what is seen in the GP, TC and LDL-C values were lower than those collected in prepuberal age in males, also when analyzing the NS and NS/LAH groups. In females, this trend was confirmed only in the NS group. Moreover, NS patients also HDL-C showed lower values in puberty.

Our cohort seems to have a lipid profile characterized by low TC values, as seen in about 50% of the NS patients with *PTPN11* mutation, low HDL levels, as seen in NS patients (in particular in *PTPN11* mutated patients), with LDL-C and HDL-C tending to decrease during puberty, more evidently in males. The temporal patterns of lipid profile were quite opposed for NS and NS/LAH, with the former generally displaying decreasing values (except for triglycerides) while NS/LAH, especially among females, showed increasing trends with age. Triglycerides showed an increasing trend with age only in NS females. We did not observe any pathological involvement glucose homeostasis in our patients.

A slightly lower BMI was also observed, without correlation to TC and cholesterol fractions. It is currently known that low BMI is a typical feature of RASopathies and overweight has a very low prevalence, probably due to the primary involvement of adipose tissue and muscle mass (9, 12).

Previous studies in patients with RASopathies have shown that body composition changes are not a reflection of the contribution of caloric and macronutrient intake when evaluating dietary habits and energy expenditure, because the results were similar to those of the general population (9).

Currently, in the literature, there are no studies that allow us to clarify the pathogenesis of the lipid profile in RASopathies, although the dysregulation of RAS-MAPK pathway in adipogenesis has been demonstrated. In fact, studies in cellular models and knockout mice revealed an important role for ERK1 and SHP2 in adipogenesis, resulting in defective lipid metabolism (2). Our results seem to support this hypothesis: in fact, patients with PTPN11 mutations have shown greater involvement of the lipid profile, with high prevalence of low lipid levels, compared to the general population.

Further studies on *in vitro* and *in vivo* experimental models are required to understand the role of the RAS-MAPK pathway in regulating lipid metabolism and how alterations in this pathway affect metabolic profiles. It will also be important to evaluate whether the global metabolic imbalance can influence other characteristic features of RASopathies such as growth, delayed puberty, heart disease, and cognitive impairment.

## Data availability statement

The original contributions presented in the study are included in the article/supplementary material. Further inquiries can be directed to the corresponding author.

## Ethics statement

The studies involving human participants were reviewed and approved by Emilia-Romagna AVEC ethics committee [internal code 119/2022/Oss/AOUBo]. Written informed consent to participate in this study was provided by the participants’ legal guardian/next of kin.

## Author contributions

FT, LM, ES, MT and AnP designed, interpreted the data, and wrote the manuscript. MS, AM and CS collected the data. DG and MZ provided the statistical analysis. FT, ES, and AnnP cared for the patients and coordinated all clinical investigation. CR carried out molecular analysis.
